# Supplementary material for: Inhibiting EZH2 targets atypical teratoid rhabdoid tumor by triggering viral mimicry via both RNA and DNA sensing pathways
Source: Nat Commun. 2024 Oct 29;15:9321. doi: 10.1038/s41467-024-53515-8 (PMC11522499; doi:10.1038/s41467-024-53515-8)
Supplement: Supplementary file 1 — Supplementary Information [file 41467_2024_53515_MOESM1_ESM.pdf]

# Inhibiting EZH2 Targets Atypical Teratoid Rhabdoid Tumor by Triggering Viral Mimicry via Both RNA and DNA Sensing Pathways

Shengrui Feng[1,2,13]*, Sajid A. Marhon[1,13], Dustin J. Sokolowski[3,4,13], Alister D'Costa[5,6], Fraser Soares[1], Parinaz Mehdipour[1], Charles Ishak[1], Helen Loo Yau[1,7], Ilias Ettayebi[1,7], Parasvi S. Patel[1,7], Raymond Chen[1, 7], Jiming Liu[8], Philip C. Zuzarte[6], King Ching Ho[9,10], Ben Ho[11], Shiyao Ning[1], Annie Huang[2,9,10,11], Cheryl H. Arrowsmith[1,7,12], Michael D. Wilson[3,4], Jared T. Simpson[5,6], Daniel D. De Carvalho[1,7]*

[1]. Princess Margaret Cancer Centre, University Health Network, Toronto, Ontario, Canada

[2]. The First Affiliated Hospital of University of South China, Hengyang, Hunan, China

[3]. Department of Molecular Genetics, University of Toronto, Toronto, Ontario, Canada

[4]. Genetics and Genome Biology, SickKids Research Institute, Toronto, Ontario, Canada

[5]. Department of Computer Science, University of Toronto, Toronto, Ontario, Canada

[6]. Ontario Institute for Cancer Research, Toronto, Ontario, Canada

[7]. Department of Medical Biophysics, University of Toronto, Toronto, Ontario, Canada

[8]. The Cardiac Development and Early Intervention Unit, West China Institute of Women and Children's Health, West China Second University Hospital, Sichuan University, Chengdu, China

[9]. Division of Hematology/Oncology, Hospital for Sick Children, Toronto, Ontario, Canada

[10]. Arthur and Sonia Labatt Brain Tumour Research Centre, Hospital for Sick Children, Toronto, Ontario, Canada

[11]. Laboratory Medicine and Pathobiology, Faculty of Medicine, University of Toronto, Ontario, Canada

[12]. Structural Genomics Consortium, University of Toronto, Toronto, Ontario, Canada

[13]. These authors contributed equally: Shengrui Feng, Sajid A. Marhon, Dustin J. Sokolowski

*Address correspondences to
Dr. Shengrui Feng, Email: shengrui.feng@uhn.ca
Dr. Daniel D. De Carvalho, Email: daniel.decarvalho@uhnresearch.ca

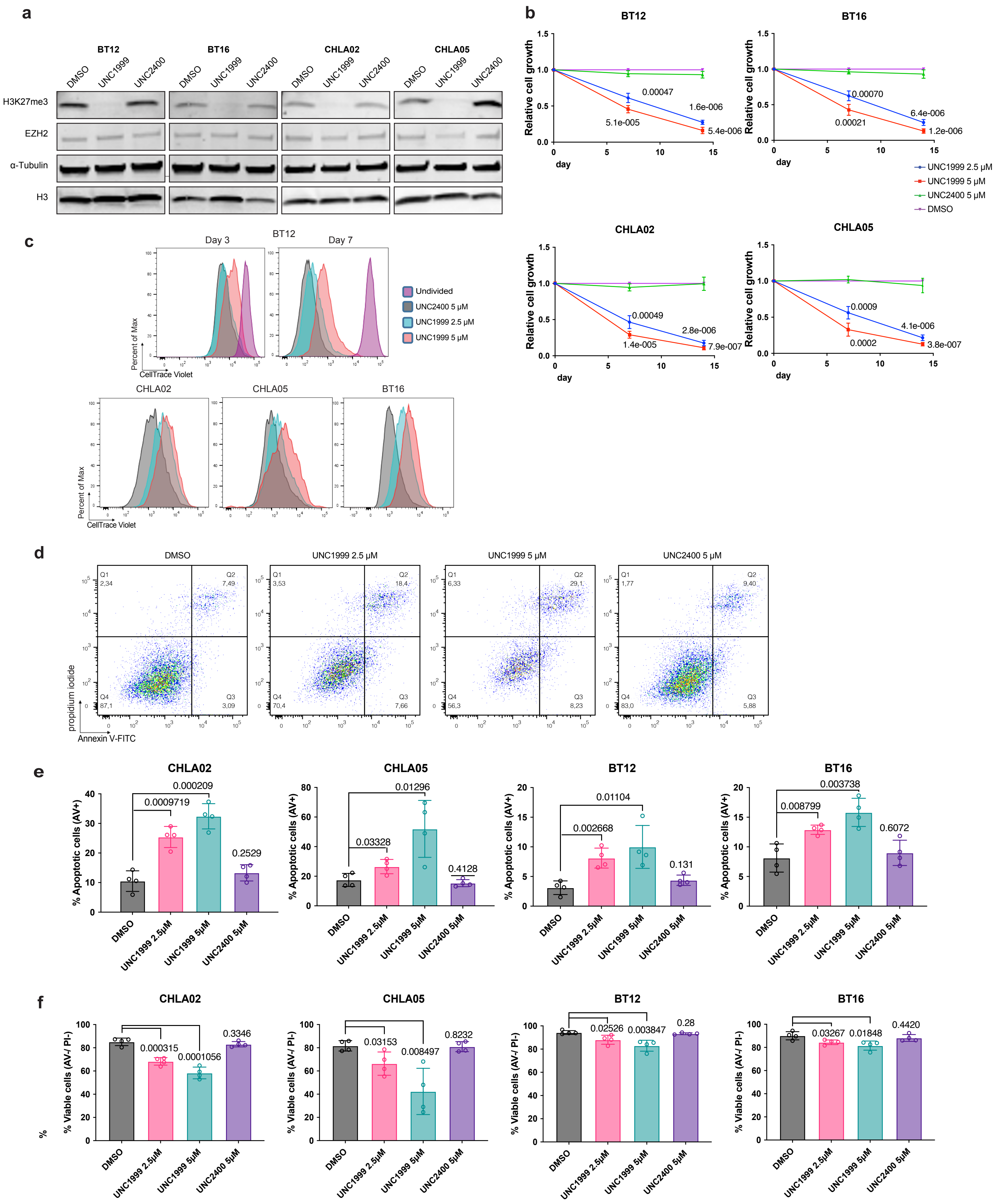

a
BT12
BT16
CHLA02
CHLA05
DMSO
UNC1999
UNC2400
H3K27me3
EZH2
α-Tubulin
H3
b
BT12
BT16
CHLA02
CHLA05
Relative cell growth
day
0.00047
1.6e-006
5.1e-005
5.4e-006
0.00070
6.4e-006
0.00021
1.2e-006
0.00049
2.8e-006
1.4e-005
7.9e-007
0.0009
4.1e-006
0.0002
3.8e-007
UNC1999 2.5 µM
UNC1999 5 µM
UNC2400 5 µM
DMSO
c
BT12
Day 3
Day 7
Percent of Max
CellTrace Violet
Undivided
UNC2400 5 µM
UNC1999 2.5 µM
UNC1999 5 µM
CHLA02
CHLA05
BT16
d
DMSO
UNC1999 2.5 µM
UNC1999 5 µM
UNC2400 5 µM
propidium iodide
Annexin V-FITC
Q1 2,34
Q2 7,49
Q4 87,1
Q3 3,09
Q1 3,53
Q2 18,4
Q4 70,4
Q3 7,66
Q1 6,33
Q2 29,1
Q4 56,3
Q3 8,23
Q1 1,77
Q2 9,40
Q4 83,0
Q3 5,88
e
CHLA02
CHLA05
BT12
BT16
% Apoptotic cells (AV+)
0.000209
0.0009719
0.2529
0.01296
0.03328
0.4128
0.01104
0.002668
0.131
0.003738
0.008799
0.6072
DMSO
UNC1999 2.5µM
UNC1999 5µM
UNC2400 5µM
f
CHLA02
CHLA05
BT12
BT16
% Viable cells (AV-/ PI-)
%
0.3346
0.000315
0.0001056
0.03153
0.008497
0.8232
0.02526
0.003847
0.28
0.03267
0.01848
0.4420
DMSO
UNC1999 2.5µM
UNC1999 5µM
UNC2400 5µM

**Supplementary Fig. 1. UNC1999 treatment impairs ATRT cell fitness.**

**a**, Representative immunoblots showing the expression level of H3K27me3, EZH2, α-Tubulin, and H3 in four indicated ATRT cell lines treated with DMSO, UNC2400, or UNC1999.

**b**, Relative cell growth was measured by counting viable cells in four indicated ATRT cell lines treated with DMSO, 5 μM UNC2400, 2.5 μM UNC1999, or 5 μM UNC1999 at day 7 and day 14. Data are mean ± SD of three biologically independent replicates; P-value is calculated by multiple unpaired t tests (two-tailed) followed by correction for multiple testing.

**c**, CellTrace Violet (CTV) proliferation analysis in ATRT cell lines treated with DMSO, UNC1999, or UNC2400.

**d**, Representative flow plots of Annexin V/PI staining in CHLA02 cells treated with DMSO, UNC1999, or UNC2400 from four independent experiments.

**e,f,** Quantification of apoptotic (AV+) fractions (**e**) and viable cell fractions (AV-/ PI-)(**f**) in ATRT cell lines treated with DMSO, UNC1999, or UNC2400. Data are mean ± SD of four biologically independent replicates; P-value is calculated by two-tailed unpaired t tests (**e, f**). Source data are provided as a Source Data file.

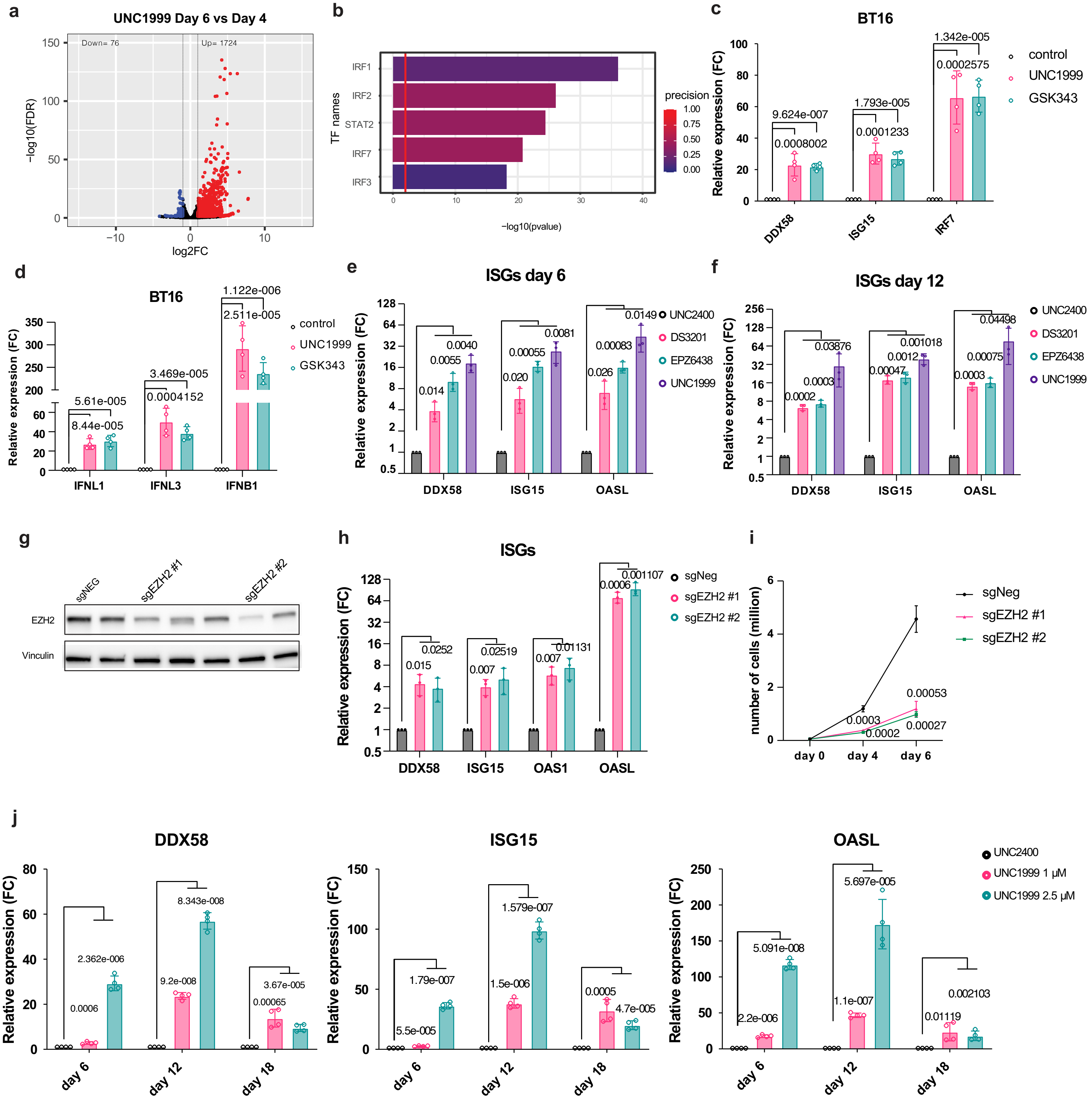

a
UNC1999 Day 6 vs Day 4
Down= 76
Up= 1724
−log10(FDR)
log2FC
b
TF names
IRF1
IRF2
STAT2
IRF7
IRF3
precision
−log10(pvalue)
c
BT16
Relative expression (FC)
control
UNC1999
GSK343
DDX58
ISG15
IRF7
d
BT16
IFNL1
IFNL3
IFNB1
e
ISGs day 6
UNC2400
DS3201
EPZ6438
UNC1999
f
ISGs day 12
g
sgNEG
sgEZH2 #1
sgEZH2 #2
EZH2
Vinculin
h
ISGs
sgNeg
sgEZH2 #1
sgEZH2 #2
DDX58
ISG15
OAS1
OASL
i
number of cells (million)
day 0
day 4
day 6
j
DDX58
ISG15
OASL
UNC1999 1 μM
UNC1999 2.5 μM
day 6
day 12
day 18

**Supplementary Fig. 2. Pharmacological or genetic inhibition of EZH2 triggers interferon signaling.**

**a**, Volcano plots showing the genes differential analysis statistics of UNC1999 day 6 versus day 4. Blue dots represent downregulated genes and red dots represent upregulated genes. Black dots represent genes that not differentially regulated. Significance was determined by $|log_2FC|$>1 and $\mathrm{FDR} < 0.05$. Negative binomial likelihood ratio test with BH (Benjamini–Hochberg)-corrected for multiple testing.

**b**, Analysis of transcription factor binding site enrichments for upregulated genes in UNC1999 day 6 versus day 4. P-value was calculated by the one-sided hypergeometric test followed by correction for multiple testing.

**c**,**d**, The expression of indicated ISGs (**c**), and IFN genes (**d**), in BT16 cells with either UNC1999 or GSK343 treatment was measured by quantitative real-time PCR at day 6.

**e**,**f**, The expression of indicated ISGs in BT16 cells treated with UNC2400, UNC1999, Valemetostat (DS-3201), or Tazemetostat (EPZ6438) was measured by quantitative real-time PCR at day 6 (**e**) and day 12 (**f**).

**g**, Representative immunoblots showing the expression level of EZH2 and Vinculin in BT16 cell line with sgRNA against negative control and EZH2.

**h**, The expression of indicated ISGs in BT16 cell line with sgRNA against negative control and EZH2 was measured by quantitative real-time. sgEZH2 #1 and sgEZH2 #1 represent two independent knockout cell lines deficient for EZH2.

**i**, Number of viable cells in culture of BT16 cell line with sgRNA against negative control and EZH2 were counted at day 0, day 4, and day 6.

**j**, The expression of indicated ISGs in BT16 cells treated with UNC2400 or UNC1999 at 1 µM or 2.5 µM was measured by quantitative real-time PCR at day 6, day 12, and day 18.

Data are mean ± SD of three (**e**, **f**, **h**, **i**) or four (**c**, **d**, **j**) biologically independent replicates; P-value is calculated by multiple unpaired t tests (two-tailed) followed by correction for multiple testing (**c**, **d**, **e**, **f**, **h**, **i**, **j**). Source data are provided as a Source Data file.

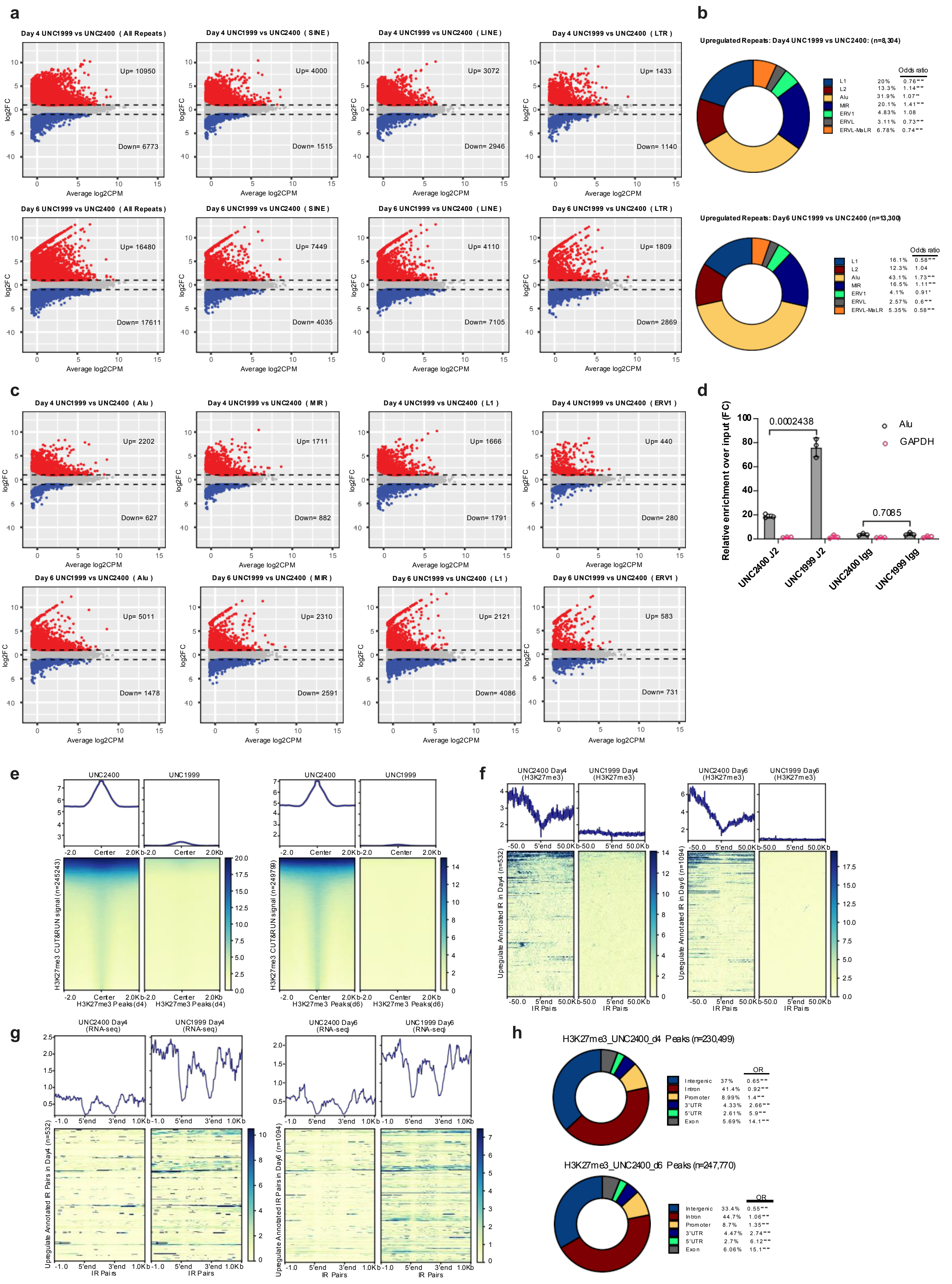
a
Day 4 UNC1999 vs UNC2400 ( All Repeats )
Up= 10950
Down= 6773
Day 4 UNC1999 vs UNC2400 ( SINE )
Up= 4000
Down= 1515
Day 4 UNC1999 vs UNC2400 ( LINE )
Up= 3072
Down= 2946
Day 4 UNC1999 vs UNC2400 ( LTR )
Up= 1433
Down= 1140
Day 6 UNC1999 vs UNC2400 ( All Repeats )
Up= 16480
Down= 17611
Day 6 UNC1999 vs UNC2400 ( SINE )
Up= 7449
Down= 4035
Day 6 UNC1999 vs UNC2400 ( LINE )
Up= 4110
Down= 7105
Day 6 UNC1999 vs UNC2400 ( LTR )
Up= 1809
Down= 2869
log2FC
Average log2CPM
b
Upregulated Repeats: Day4 UNC1999 vs UNC2400: (n=8,304)
Odds ratio
L1 20% 0.76***
L2 13.3% 1.14***
Alu 31.9% 1.07**
MIR 20.1% 1.41***
ERV1 4.83% 1.08
ERVL 3.11% 0.73***
ERVL-MaLR 6.78% 0.74***
Upregulated Repeats: Day6 UNC1999 vs UNC2400 (n=13,300)
Odds ratio
L1 16.1% 0.58***
L2 12.3% 1.04
Alu 43.1% 1.73***
MIR 16.5% 1.11***
ERV1 4.1% 0.91*
ERVL 2.57% 0.6***
ERVL-MaLR 5.35% 0.58***
c
Day 4 UNC1999 vs UNC2400 ( Alu )
Up= 2202
Down= 627
Day 4 UNC1999 vs UNC2400 ( MIR )
Up= 1711
Down= 882
Day 4 UNC1999 vs UNC2400 ( L1 )
Up= 1666
Down= 1791
Day 4 UNC1999 vs UNC2400 ( ERV1 )
Up= 440
Down= 280
Day 6 UNC1999 vs UNC2400 ( Alu )
Up= 5011
Down= 1478
Day 6 UNC1999 vs UNC2400 ( MIR )
Up= 2310
Down= 2591
Day 6 UNC1999 vs UNC2400 ( L1 )
Up= 2121
Down= 4086
Day 6 UNC1999 vs UNC2400 ( ERV1 )
Up= 583
Down= 731
log2FC
Average log2CPM
d
Relative enrichment over input (FC)
0.0002438
0.7085
Alu
GAPDH
UNC2400 J2
UNC1999 J2
UNC2400 Igg
UNC1999 Igg
e
UNC2400
UNC1999
H3K27me3 CUT&RUN signal (n=245243)
H3K27me3 Peaks(d4)
H3K27me3 CUT&RUN signal (n=249799)
H3K27me3 Peaks(d6)
-2.0 Center 2.0Kb
f
UNC2400 Day4 (H3K27me3)
UNC1999 Day4 (H3K27me3)
UNC2400 Day6 (H3K27me3)
UNC1999 Day6 (H3K27me3)
Upregulate Annotated IR in Day4 (n=532)
Upregulate Annotated IR in Day6 (n=1094)
-50.0 5'end 50.0Kb
IR Pairs
g
UNC2400 Day4 (RNA-seq)
UNC1999 Day4 (RNA-seq)
UNC2400 Day6 (RNA-seq)
UNC1999 Day6 (RNA-seq)
Upregulate Annotated IR Pairs in Day4 (n=532)
Upregulate Annotated IR Pairs in Day6 (n=1094)
-1.0 5'end 3'end 1.0Kb
IR Pairs
h
H3K27me3_UNC2400_d4 Peaks (n=230,499)
OR
Intergenic 37% 0.65***
Intron 41.4% 0.92***
Promoter 8.99% 1.4***
3'UTR 4.33% 2.66***
5'UTR 2.61% 5.9***
Exon 5.69% 14.1***
H3K27me3_UNC2400_d6 Peaks (n=247,770)
OR
Intergenic 33.4% 0.55***
Intron 44.7% 1.06***
Promoter 8.7% 1.35***
3'UTR 4.47% 2.74***
5'UTR 2.7% 6.12***
Exon 6.06% 15.1***

**Supplementary Fig. 3. UNC1999 treatment triggers RNA sensing pathway through derepressing Alu IRs.**

**a**, MA plots showing $\log_2$FC versus Average $\log_2$CPM for differential analysis of repetitive elements of UNC1999 versus UNC2400 at day 4 (top row) and day 6 (bottom row). Plots from left to right are for all repeat classes, SINE, LINE, and LTR class. Red and blue dots represent the upregulated and downregulated repeat elements respectively. Gray dots represent repeats that are not differentially regulated. Significance was determined by $|log_2FC|>1$, and $\mathrm{FDR}<0.05$. Negative binomial likelihood ratio test with BH (Benjamini–Hochberg)-corrected for multiple testing.

**b**, Donut plots showing the distribution of the upregulated major repeat families in UNC1999 versus UNC2400 at day 4 (left) and day 6 (right). Counts of the upregulated repeat families were compared with the whole genome counts using two-sided Fisher exact test to calculate the odds ratio and p-value. *$p<0.05$; **$p<0.01$; ***$p<0.001$; ****$p<0.0001$. The p-values for day 4 plot (top) are <2.2e-16 for L1, 7.80e-05 for L2, 7.0e-03 for Alu, <2.2e-16 for MIR, 0.31 for ERV1, 3.27e-07 for ERVL and 9.68e-13 for ERVL-MaLR. The p-values for day 6 plot (bottom) are <2.2e-16 for L1, 0.14 for L2, <2.2e-16 for Alu, 1.23e-05 for MIR, 3.0e-02 for ERV1, and <2.2e-16 for ERVL and ERVL-MaLR.

**c**, MA plots showing $\log_2$FC versus Average $\log_2$CPM for differential analysis of major repeat families of UNC1999 versus UNC2400 at day 4 (top row) and day 6 (bottom row). Plots from left to right are for Alu, MIR, L1 and ERV1 family. Red and blue dots represent the upregulated and downregulated repeat elements respectively. Gray dots represent repeat elements that are not differentially regulated. Significance was determined by $|log_2FC|>1$, and $\mathrm{FDR}<0.05$. Negative binomial likelihood ratio test with BH (Benjamini–Hochberg)-corrected for multiple testing.

**d**, Error bar plots showing the enrichment of transcripts of Alu repeats or GAPDH in the dsRNA species immunoprecipitated with J2 antibody from total RNA harvested from UNC1999 or UNC2400 treated BT16 cells. qRT-PCR was employed for analysis, with normalization to the corresponding input RNA. Data are mean ± SD of three biologically independent replicates; P-value is calculated by unpaired t tests (two-tailed).

**e**, Heatmap and average profile of the H3K27me3 CUT&RUN signal in samples treated with UNC1999 or UNC2400 at pooled peaks of the two conditions with -/+2Kb up/downstream of the center of the peaks in UNC1999 versus UNC2400 day 4 (left) and day 6 (right).

**f,g**, Heatmap and average profile showing the RNA-seq signal at upregulated Annotated IR pairs in UNC1999 versus UNC2400 in Day4 (**f**) and Day6 (**g**). Signal tracks were plotted 1 Kb upstream the 5'end of the IR pairs and 1Kb downstream the 3' end of the IR pairs. The orientation and the strand are based on the RNA-seq transcriptional orientation of the IR pairs.

**h,** Donut plots showing the distribution of the H3K27me3 peaks in samples treated with UNC2400 at day 4 (top) and day 6 (bottom). Odds ratio and p-value were calculated using the Fisher exact test by comparing the counts of the peaks at the genomic regions with the whole human genome annotation. ****$p<0.0001$. The p-value for day 4 plot (top) is <2.2e-16 for Intergenic, Intron, Promoter, 3' UTR, 5' UTR, and Exon. The p-value for the day 6 plot (bottom) is <2.2e-16 for Intergenic, Intron, Promoter, 3' UTR, 5' UTR, and Exon.

Source data are provided as a Source Data file.

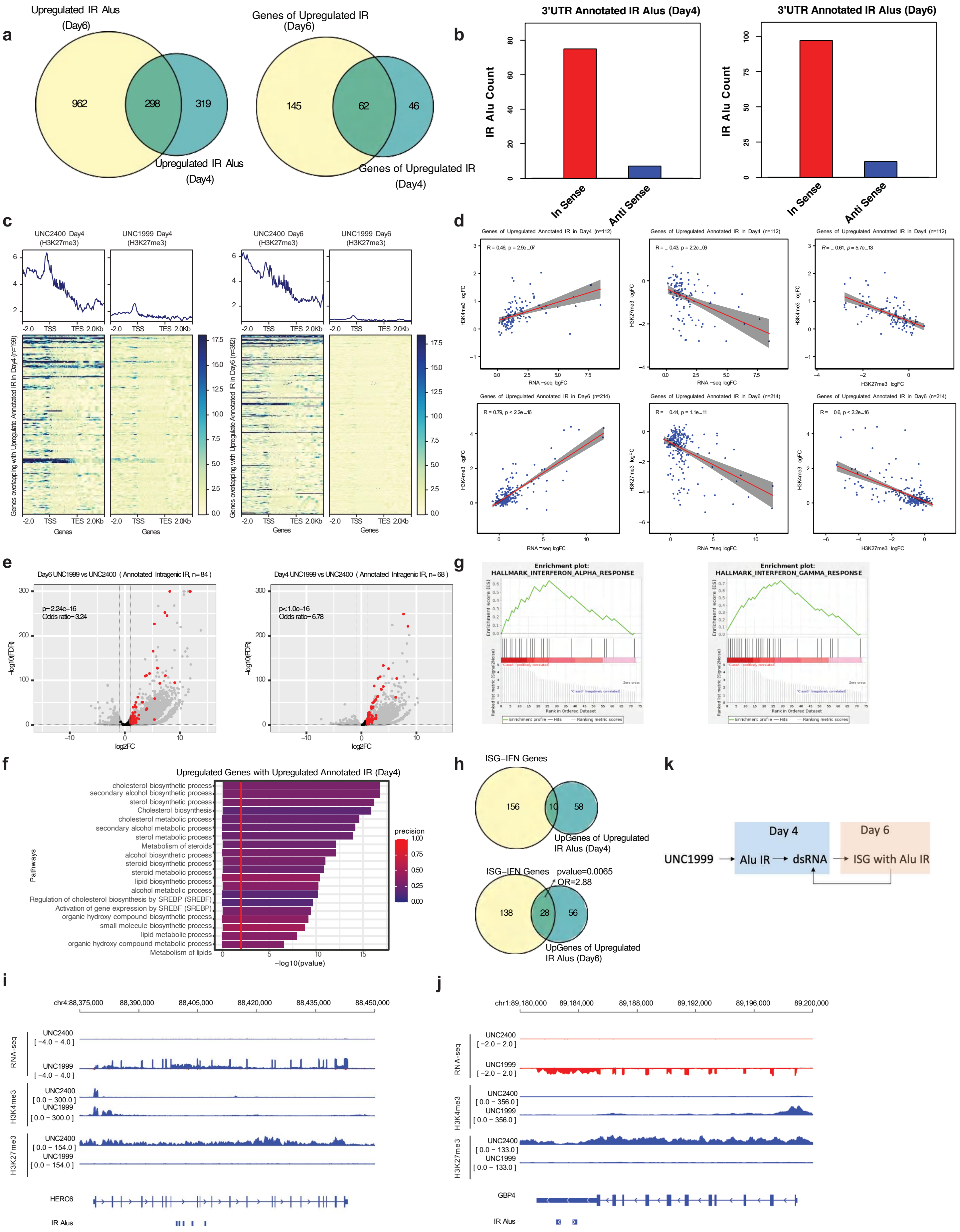
a
Upregulated IR Alus (Day6)
962
298
319
Upregulated IR Alus (Day4)
Genes of Upregulated IR (Day6)
145
62
46
Genes of Upregulated IR (Day4)
b
3'UTR Annotated IR Alus (Day4)
3'UTR Annotated IR Alus (Day6)
IR Alu Count
In Sense
Anti Sense
c
UNC2400 Day4 (H3K27me3)
UNC1999 Day4 (H3K27me3)
UNC2400 Day6 (H3K27me3)
UNC1999 Day6 (H3K27me3)
Genes overlapping with Upregulate Annotated IR in Day4 (n=199)
Genes overlapping with Upregulate Annotated IR in Day6 (n=382)
-2.0 TSS TES 2.0Kb
Genes
d
Genes of Upregulated Annotated IR in Day4 (n=112)
R = 0.46, p = 2.9e−07
R = − 0.43, p = 2.2e−06
R = − 0.61, p = 5.7e−13
Genes of Upregulated Annotated IR in Day6 (n=214)
R = 0.79, p < 2.2e−16
R = − 0.44, p = 1.1e−11
R = − 0.6, p < 2.2e−16
H3K4me3 logFC
H3K27me3 logFC
RNA−seq logFC
e
Day6 UNC1999 vs UNC2400 ( Annotated Intragenic IR, n= 84 )
p=2.24e−16
Odds ratio=3.24
Day4 UNC1999 vs UNC2400 ( Annotated Intragenic IR, n= 68 )
p< 1.0e−16
Odds ratio= 6.78
−log10(FDR)
log2FC
g
Enrichment plot: HALLMARK_INTERFERON_ALPHA_RESPONSE
Enrichment plot: HALLMARK_INTERFERON_GAMMA_RESPONSE
Rank in Ordered Dataset
f
Upregulated Genes with Upregulated Annotated IR (Day4)
cholesterol biosynthetic process
secondary alcohol biosynthetic process
sterol biosynthetic process
Cholesterol biosynthesis
cholesterol metabolic process
secondary alcohol metabolic process
sterol metabolic process
Metabolism of steroids
alcohol biosynthetic process
steroid biosynthetic process
steroid metabolic process
lipid biosynthetic process
alcohol metabolic process
Regulation of cholesterol biosynthesis by SREBP (SREBF)
Activation of gene expression by SREBF (SREBP)
organic hydroxy compound biosynthetic process
small molecule biosynthetic process
lipid metabolic process
organic hydroxy compound metabolic process
Metabolism of lipids
Pathways
precision
−log10(pvalue)
h
ISG−IFN Genes
156
10
58
UpGenes of Upregulated IR Alus (Day4)
ISG−IFN Genes
pvalue=0.0065
OR=2.88
138
28
56
UpGenes of Upregulated IR Alus (Day6)
k
Day 4
Day 6
UNC1999
Alu IR
dsRNA
ISG with Alu IR
i
chr4:88,375,000
88,390,000
88,405,000
88,420,000
88,435,000
88,450,000
RNA-seq
H3K4me3
H3K27me3
UNC2400
UNC1999
HERC6
IR Alus
j
chr1:89,180,000
89,184,000
89,188,000
89,192,000
89,196,000
89,200,000
GBP4
IR Alus

**Supplementary Fig. 4. UNC1999 induces intronic transcription that expresses IR-Alu elements.**
**a**, Venn diagrams showing the overlap between the lists of Annotated IR-Alu elements that are upregulated in UNC1999 versus UNC2400 day 4 and those that are upregulated in UNC1999 versus UNC2400 day 6 (left), and the overlap between sets of genes overlapping with those upregulated IR-Alu elements in day 4 and day 6 (right).
**b**, Counts of the upregulated 3'UTR Annotated IR-Alu elements at day 4 (left, *n*=82) and day 6 (right, *n*=108) that have transcription in sense and anti-sense of their overlapping genes.
**c**, Heatmap and average profile of the H3K27me3 CUT&RUN signal in UNC1999 and UNC2400 samples at the gene body with -/+2Kb up/downstream of genes that overlap with upregulated IRs in UNC1999 versus UNC2400 day 4 (left) and day 6 (right).
**d**, Scatter plots correlating the $\log_2 FC$ of H3K4me3 with RNA-seq (left), H3K27me3 with RNA-seq (middle), and H3K4me3 with H3K27me3 (right) at day 4 (top row, *n*=112) and day 6 (bottom row, *n*=214) of genes with upregulated annotated IRs pairs in the 3'UTR and intronic regions. FC for the histone marks was calculated as the ratio of the UNC1999 to UNC2400 CUT&RUN signal at +/- 5Kb of the TSS of the gene, while FC for the RNA-seq was calculated as the ratio of the RNA-seq count per million (CPM) of gene body in UNC1999 to UNC2400. R represents the Pearson correlation coefficient, and p-values were calculated using the two-sided t test.
**e**, Volcano plots showing genes differential analysis statistics with highlighting dots (red and black dots) that represent genes overlapping with upregulated Annotated IR-Alu elements in day 4 UNC1999 versus UNC2400 (left) and day 6 UNC1999 versus UNC2400 (right). Upregulation was determined by $\log_2 FC > 1$ and $FDR < 0.05$. Counts of genes overlapping with IR and are upregulated at day 4 (*n*=68) and day 6 (*n*=84) were compared with the counts of all upregulated genes using two-sided Fisher exact test to calculate the odds ratio and p-value.
**f**, Pathway analysis of upregulated genes (*n*=68) that overlap with upregulated intragenic IRs in UNC1999 versus UNC2400 at day 4. P-value is calculated by the one-sided hypergeometric test followed by correction for multiple testing.
**g**, Gene set enrichment plots of the enriched pathways in set of upregulated genes overlapping with upregulated IR pairs in day 6 UNC1999 versus UNC2400.
**h,** Venn diagram showing the intersection between the union set of the ISG genes and IFN pathway genes with the set of upregulated genes that overlap with upregulated IR-Alus in UNC1999 versus UNC2400-treated samples at day 4 (top row) and day 6 (bottom row). The count of upregulated ISG genes overlapping with upregulated IR Alus at day 6 was compared to that at day 4 using the one-tailed Fisher exact test to calculate the odds ratio and p-value.
**i**,**j** Genome track signal of the RNA-seq and of H3K4me3 and K3K27me3 CUT&RUN marks at HERC6 (**i**) and GBP4 (**j**) loci in day 6 UNC1999 and UNC2400 samples. HERC6 and GBP4 genes are significantly upregulated in day 6 UNC1999 versus UNC2400. The plots include a track for upregulated IR-Alu elements overlapping with the HERC6 and GBP4 genes. RNA-seq signal was plotted from the two strands separately. The RNA-seq forward strand signal was plotted in blue and positive range, while the RNA-seq reverse strand signal was plotted in red and negative range. RNA-seq tracks are plotted on a log scale. The log scale of the forward strand signal was calculated as $log(signaltrack+1)$, while the log scale of the reverse strand signal was calculated as $-log(-signaltrack+1)$.
**k**, Schematic representation of a feedback loop in which ISG's have Alu-IR sequences that reinforce the viral response mechanism.

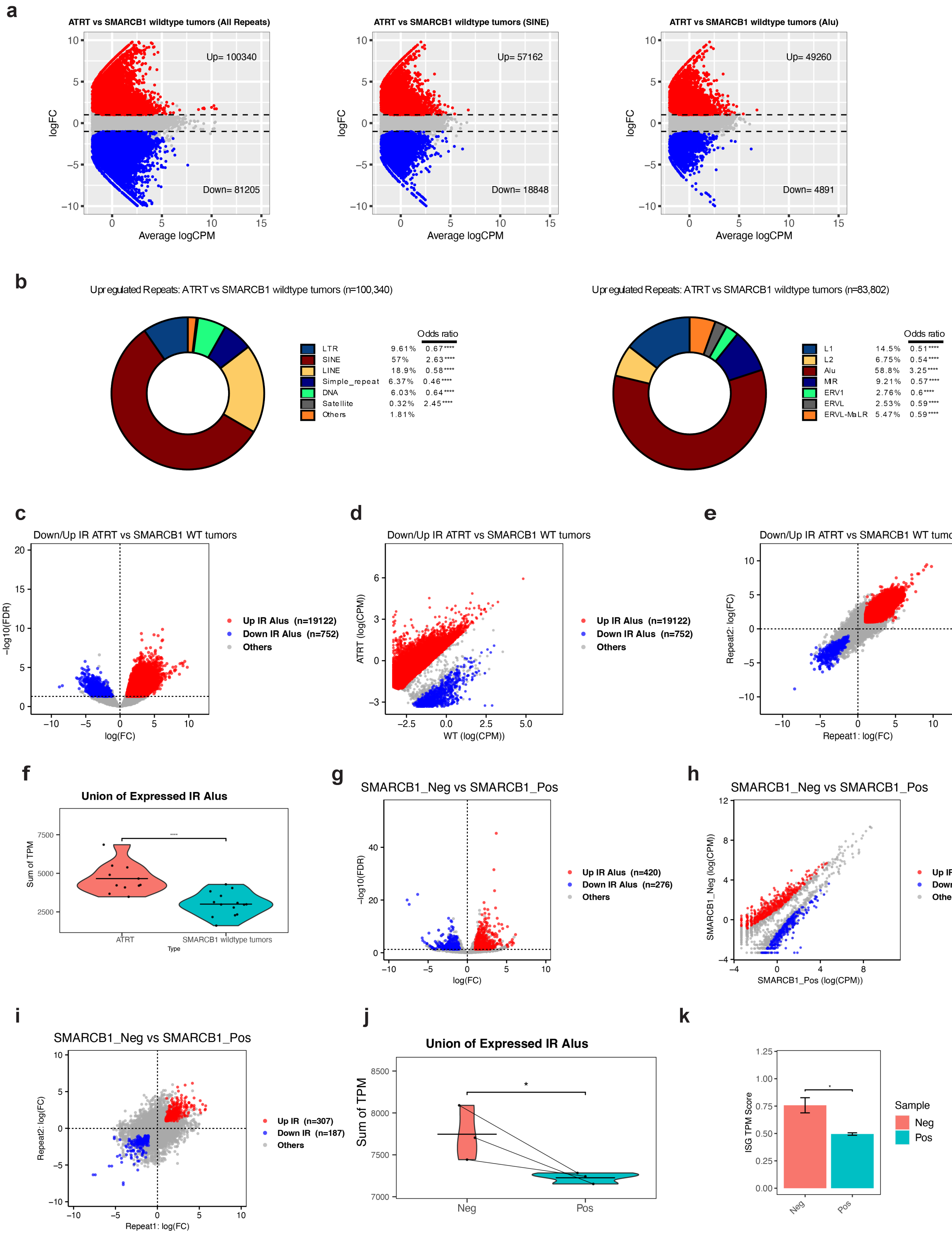
a
ATRT vs SMARCB1 wildtype tumors (All Repeats)
Up= 100340
Down= 81205
ATRT vs SMARCB1 wildtype tumors (SINE)
Up= 57162
Down= 18848
ATRT vs SMARCB1 wildtype tumors (Alu)
Up= 49260
Down= 4891
logFC
Average logCPM
b
Upregulated Repeats: ATRT vs SMARCB1 wildtype tumors (n=100,340)
Odds ratio
LTR 9.61% 0.67****
SINE 57% 2.63****
LINE 18.9% 0.58****
Simple_repeat 6.37% 0.46****
DNA 6.03% 0.64****
Satellite 0.32% 2.45****
Others 1.81%
Upregulated Repeats: ATRT vs SMARCB1 wildtype tumors (n=83,802)
Odds ratio
L1 14.5% 0.51****
L2 6.75% 0.54****
Alu 58.8% 3.25****
MIR 9.21% 0.57****
ERV1 2.76% 0.6****
ERVL 2.53% 0.59****
ERVL-MaLR 5.47% 0.59****
c
Down/Up IR ATRT vs SMARCB1 WT tumors
−log10(FDR)
log(FC)
Up IR Alus (n=19122)
Down IR Alus (n=752)
Others
d
Down/Up IR ATRT vs SMARCB1 WT tumors
ATRT (log(CPM))
WT (log(CPM))
Up IR Alus (n=19122)
Down IR Alus (n=752)
Others
e
Down/Up IR ATRT vs SMARCB1 WT tumors
Repeat2: log(FC)
Repeat1: log(FC)
Up IR (n=17013)
Down IR (n=445)
Others
f
Union of Expressed IR Alus
Sum of TPM
****
ATRT
SMARCB1 wildtype tumors
Type
g
SMARCB1_Neg vs SMARCB1_Pos
−log10(FDR)
log(FC)
Up IR Alus (n=420)
Down IR Alus (n=276)
Others
h
SMARCB1_Neg vs SMARCB1_Pos
SMARCB1_Neg (log(CPM))
SMARCB1_Pos (log(CPM))
Up IR Alus (n=420)
Down IR Alus (n=276)
Others
i
SMARCB1_Neg vs SMARCB1_Pos
Repeat2: log(FC)
Repeat1: log(FC)
Up IR (n=307)
Down IR (n=187)
Others
j
Union of Expressed IR Alus
Sum of TPM
*
Neg
Pos
k
ISG TPM Score
*
Sample
Neg
Pos

**Supplementary Fig. 5. SMARCB1 loss primes ATRTs for viral mimicry induction**

**a,** Mean-average (MA) plots showing the upregulated and downregulated repeats in ATRT samples (*n*=11) versus SMARCB1-wildtype tumors (*n*=14) for all repeat, SINE and Alu elements. The *x* axis depicts the average of $\log_2(\mathrm{CPM})$ in all samples and the *y* axis depicts $\log_2(\mathrm{FC})$. The red dots represent the upregulated repeat element which have $\log_2(\mathrm{FC})>1$ and $\mathrm{FDR}<0.05$, while the blue dots represent the downregulated repeat elements which have $\log_2(\mathrm{FC})<1$ and $\mathrm{FDR}<0.05$. Gray dots represent the repeat elements that are not significantly regulated.

**b,** Donut plots showing the percent of repeat classes (left) or families (right) upregulated in ATRTs when compared with SMARCB1-wildtype tumors. Odds ratio and p-value are calculated using two-sided Fisher exact test by comparing the element counts of upregulated classes/families compared to the expected genomic distribution. ****$p<0.0001$. The p-value of the repeat classes plot (left) is <2.2e-16 for LTR, SINE, LINE, Simple repeat, DNA, and Satellite. The p-value of the major families (right) is <2.2e-16 for L1, L2, Alu, MIR, ERV1, ERVL, and ERVL-MaLR.

**c,** Volcano plot showing $\log_{10}(\mathrm{FDR})$ versus $\log_2(\mathrm{FC})$ of upregulated (red dots) and downregulated (blue dots) IR-Alus in ATRTs (*n*=11) compared with SMARCB1-wildtype tumors (*n*=14).

**d,** Scatter plot showing the average of $\log(\mathrm{CPM})$ values of the upregulated (red dots) and downregulated (blue dots) IR-Alus in ATRT samples versus SMARCB1-wildtype tumors.

**e,** Scatter plot showing the $\log_2(\mathrm{FC})$ of upregulated/downregulated IR-Alu pairs. The *x* axis depicts the $\log_2(\mathrm{FC})$ of one Alu, while the *y* axis depicts the $\log_2(\mathrm{FC})$ of the other Alu. An IR-Alu pair is considered differentially regulated when both Alus exhibit $|\log_2(\mathrm{FC})|>1$ and $\mathrm{FDR}<0.05$.

**f,** Violin plot showing the sum of TPM values for IR-Alus (CPM≥5) in ATRTs versus SMARCB1-wildtype tumors. P-value was calculated using the Wilcoxon rank-sum test.

**g,** Volcano plot showing $\log_{10}(\mathrm{FDR})$ versus $\log_2(\mathrm{FC})$ of upregulated and downregulated IR-Alu elements in SMARCB1 negative (*n*=3) versus SMARCB1 positive (*n*=3) I2A cell line samples at day 2. Red dots represent the upregulated IR-Alu elements which have $\log_2(\mathrm{FC})>1$ and $\mathrm{FDR}<0.05$, while blue dots represent downregulated IR-Alu elements which have $\log_2(\mathrm{FC})<1$ and $\mathrm{FDR}<0.05$.

**h,** Scatter plot showing $\log(\mathrm{CPM})$ of the upregulated and downregulated Alu elements in SMARCB1 negative (*n*=3) versus SMARCB1 positive samples (*n*=3). The *x* and *y* axes represent the mean of $\log(\mathrm{CPM})$ of the elements in SMARCB1 negative and positive samples respectively. Differentially regulated IR-Alu elements are determined with $|\log_2(\mathrm{FC})|>1$ and $\mathrm{FDR}<0.05$.

**i,** Scatter plot shows the upregulated (red dots) and downregulated (blue dots) IR-Alu pairs in SMARCB1 negative versus SMARCB1 positive samples at day 2. Gray dots represent IR-Alu pairs that are not differentially regulated. The *x* axis depicts the $\log_2(\mathrm{FC})$ of one Alu in the pair, and the *y* axis depicts the $\log_2(\mathrm{FC})$ of the other Alu in the pair. An IR-Alu pair is considered differentially regulated when each of the two Alus have $|\log_2(\mathrm{FC})|>1$ and $\mathrm{FDR}<0.05$.

**j,** Violin plot showing the sum of the TPM values of the union list of the IR-Alus with $\mathrm{CPM}\geq 5$ in each sample of the SMARCB1 negative versus SMARCB1 positive samples at day 2. The sum of the TPM values was calculated for the union list of IR-Alus in each sample. Lines connect the paired samples. *$p<0.05$ (one-tailed paired t-test).

**k,** Error bar plot comparing ISG TPM scores between SMARCB1-negative and positive samples on day 2. Scores were normalized by the mean TPM values across all samples, with each sample's score representing the mean of 38 ISG genes. *$p<0.05$ (one-tailed paired t-test).

Negative binomial likelihood ratio test with BH (Benjamini–Hochberg)-corrected for multiple testing (**a, c, e, g, h, i**).

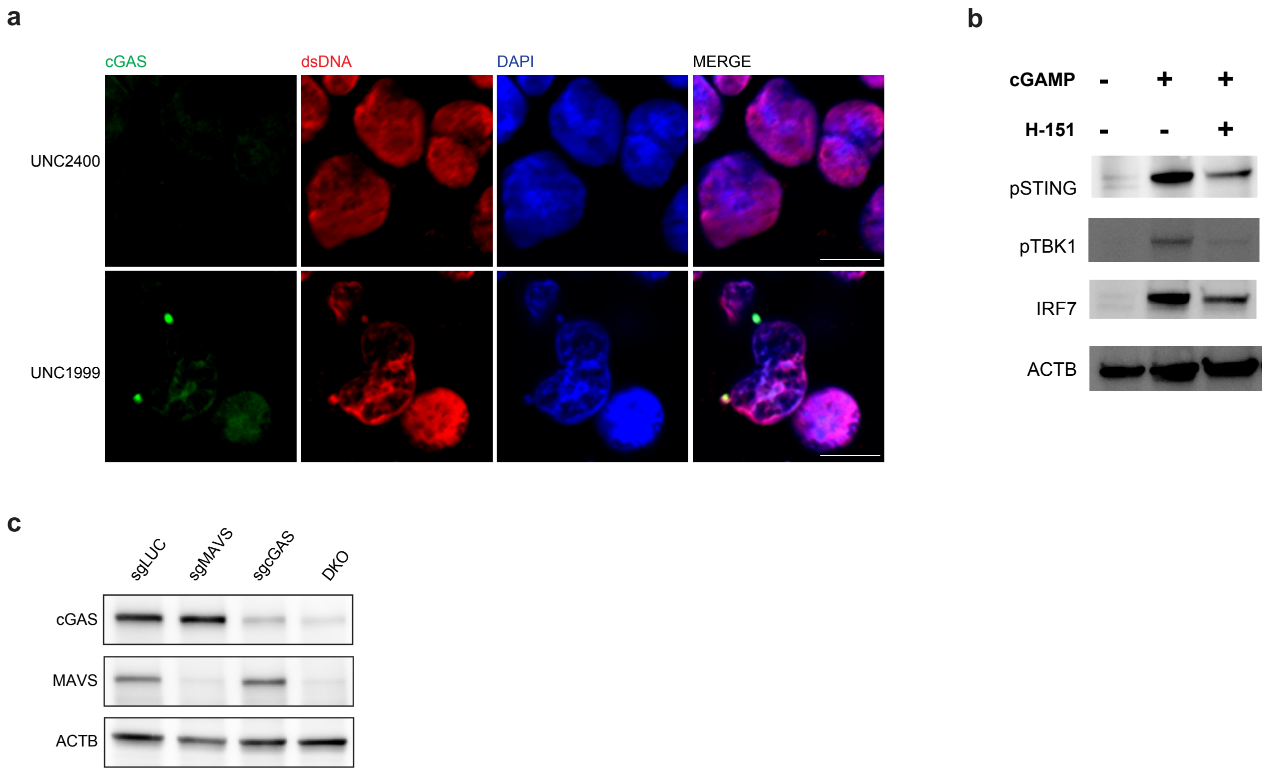
a
cGAS
dsDNA
DAPI
MERGE
UNC2400
UNC1999
b
cGAMP - + +
H-151 - - +
pSTING
pTBK1
IRF7
ACTB
c
sgLUC
sgMAVS
sgcGAS
DKO
cGAS
MAVS
ACTB

**Supplementary Fig. 6. UNC1999 treatment activates cGAS-STING DNA sensing pathway.**

**a**, Confocal microscopy of anti-cGAS and anti-dsDNA immunofluorescence in BT16 cells treated with either UNC2400 or UNC1999. cGAS was stained in green, dsDNA was stained in red, and nuclei were stained in blue (DAPI). Scale bars, 10 µm.

**b**, Representative immunoblots showing the expression level of pSTING, pTBK1 and IRF7 in THP1 cell line transfected with cGAMP in the presence or absence of H-151.

**c**, Representative immunoblots showing the expression level of MAVS and cGAS in BT16 cell line with sgRNA against LUC, MAVS, cGAS, or both cGAS and MAVS.

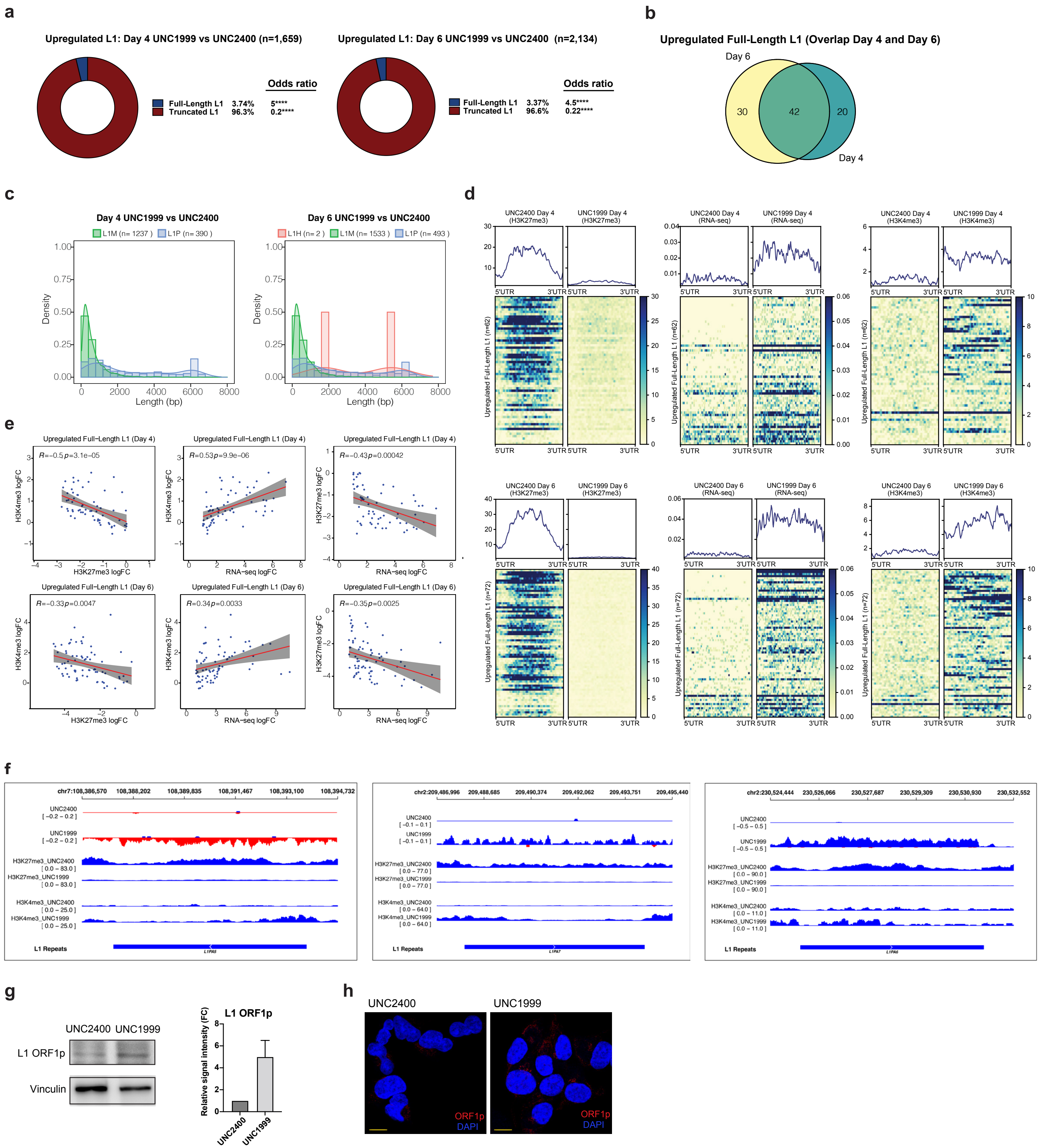
a
Upregulated L1: Day 4 UNC1999 vs UNC2400 (n=1,659)
Odds ratio
Full-Length L1 3.74% 5****
Truncated L1 96.3% 0.2****
Upregulated L1: Day 6 UNC1999 vs UNC2400 (n=2,134)
Odds ratio
Full-Length L1 3.37% 4.5****
Truncated L1 96.6% 0.22****
b
Upregulated Full-Length L1 (Overlap Day 4 and Day 6)
Day 6
Day 4
30
42
20
c
Day 4 UNC1999 vs UNC2400
L1M (n= 1237 ) L1P (n= 390 )
Day 6 UNC1999 vs UNC2400
L1H (n= 2 ) L1M (n= 1533 ) L1P (n= 493 )
Density
Length (bp)
d
UNC2400 Day 4 (H3K27me3)
UNC1999 Day 4 (H3K27me3)
UNC2400 Day 4 (RNA-seq)
UNC1999 Day 4 (RNA-seq)
UNC2400 Day 4 (H3K4me3)
UNC1999 Day 4 (H3K4me3)
Upregulated Full-Length L1 (n=62)
UNC2400 Day 6 (H3K27me3)
UNC1999 Day 6 (H3K27me3)
UNC2400 Day 6 (RNA-seq)
UNC1999 Day 6 (RNA-seq)
UNC2400 Day 6 (H3K4me3)
UNC1999 Day 6 (H3K4me3)
Upregulated Full-Length L1 (n=72)
5'UTR
3'UTR
e
Upregulated Full−Length L1 (Day 4)
R=−0.5 p=3.1e−05
R=0.53 p=9.9e−06
R=−0.43 p=0.00042
Upregulated Full−Length L1 (Day 6)
R=−0.33 p=0.0047
R=0.34 p=0.0033
R=−0.35 p=0.0025
H3K4me3 logFC
H3K27me3 logFC
RNA−seq logFC
f
chr7:108,386,570 108,388,202 108,389,835 108,391,467 108,393,100 108,394,732
UNC2400 [ -0.2 - 0.2 ]
UNC1999 [ -0.2 - 0.2 ]
H3K27me3_UNC2400 [ 0.0 - 83.0 ]
H3K27me3_UNC1999 [ 0.0 - 83.0 ]
H3K4me3_UNC2400 [ 0.0 - 25.0 ]
H3K4me3_UNC1999 [ 0.0 - 25.0 ]
L1 Repeats
L1PA5
chr2:209,486,996 209,488,685 209,490,374 209,492,062 209,493,751 209,495,440
UNC2400 [ -0.1 - 0.1 ]
UNC1999 [ -0.1 - 0.1 ]
H3K27me3_UNC2400 [ 0.0 - 77.0 ]
H3K27me3_UNC1999 [ 0.0 - 77.0 ]
H3K4me3_UNC2400 [ 0.0 - 64.0 ]
H3K4me3_UNC1999 [ 0.0 - 64.0 ]
L1 Repeats
L1PA7
chr2:230,524,444 230,526,066 230,527,687 230,529,309 230,530,930 230,532,552
UNC2400 [ -0.5 - 0.5 ]
UNC1999 [ -0.5 - 0.5 ]
H3K27me3_UNC2400 [ 0.0 - 90.0 ]
H3K27me3_UNC1999 [ 0.0 - 90.0 ]
H3K4me3_UNC2400 [ 0.0 - 11.0 ]
H3K4me3_UNC1999 [ 0.0 - 11.0 ]
L1 Repeats
L1PA6
g
UNC2400 UNC1999
L1 ORF1p
Vinculin
L1 ORF1p
Relative signal intensity (FC)
UNC2400
UNC1999
h
UNC2400
UNC1999
ORF1p
DAPI

**Supplementary Fig. 7. The expression of full-length L1s induced by UNC1999 treatment.**

**a**, Donut plots showing the proportions of truncated and full-length LINE-1 elements which are upregulated in UNC1999 versus UNC2400 at day 4 (left) and day 6 (right). Truncated and full-length elements were decided based on the 6 Kb cut-off. Counts of truncated and full-length LINE-1 elements were compared with the counts of truncated and full-length LINE-1 elements in the whole genome to calculate the odds ratio. Calculating the odds ratio and p-value was done by comparing the counts using two-sided Fisher exact test. ****$p<0.0001$. The p-value of the day 4 plot (left) is <2.2e-16 for Long L1 and Short L1. The p-value of the day 6 plot (right) is <2.2e-16 for Long L1 and Short L1.

**b**, Venn diagram showing the overlap between the sets of full-length LINE-1 elements (>6Kb) that are upregulated in UNC1999 versus UNC2400 at day 4 and day 6.

**c**, Histograms showing the length distribution of LINE-1 subfamilies that are upregulated in UNC1999 versus UNC2400 at day 4 (left) and day 6 (right).

**d**, Heatmaps and average profiles of the H3K27me3 CUT&RUN (left), RNA-seq (middle) and H3K4me3 CUT&RUN (right) plotted from UN1999 and UNC2400 samples for full-length LINE-1 elements that are upregulated in UNC1999 versus UNC2400 in day 4 (top row) and day 6 (bottom row). Signals plotted from the 5'UTR to the 3'UTR of LINE-1 elements.

**e**, Scatter plots showing the correlation between the $\log_2 FC$ of H3K4me3 CUT&RUN and H3K27me3 CUT&RUN (left), H3K4me3 CUT&RUN and RNA-seq (middle), and H3K27me3 CUT&RUN and RNA-seq (right) in day 4 (top row, $n=62$) and day 6 (bottom row, $n=72$) of upregulated full-length LINE-1 elements. FC for the histone marks was calculated as the ratio of the UNC1999 to UNC2400 CUT&RUN signal at +/- 5 Kb of the 5'UTR of upregulated LINE-1 elements, while FC for the RNA-seq was calculated as the ratio of the RNA-seq count per million (CPM) of LINE-1 elements in UNC1999 to UNC2400. R represents the Pearson correlation coefficient, and p-values were calculated using a t test.

**f**, Genome track signal of the RNA-seq and of H3K4me3 and K3K27me3 CUT&RUN marks at representative upregulated full-length LINE-1 elements from samples treated with UN1999 or UNC2400.

**g**, Representative immunoblots showing the expression level of L1 ORF1p (left) and the quantification of relative signal intensity of L1 ORF1p using ImageJ (right) in BT16 cells treated with either UNC2400 or UNC1999. Vinculin was used as a loading control. Data are mean ± SD of of two biologically independent experiments.

**h**, Confocal microscopy of anti-L1 ORF1p immunofluorescence in BT16 cells treated with either UNC2400 or UNC1999. L1 ORF1p was stained in red, and nuclei were stained in blue (DAPI). Scale bars, 10 µm.

Source data are provided as a Source Data file.

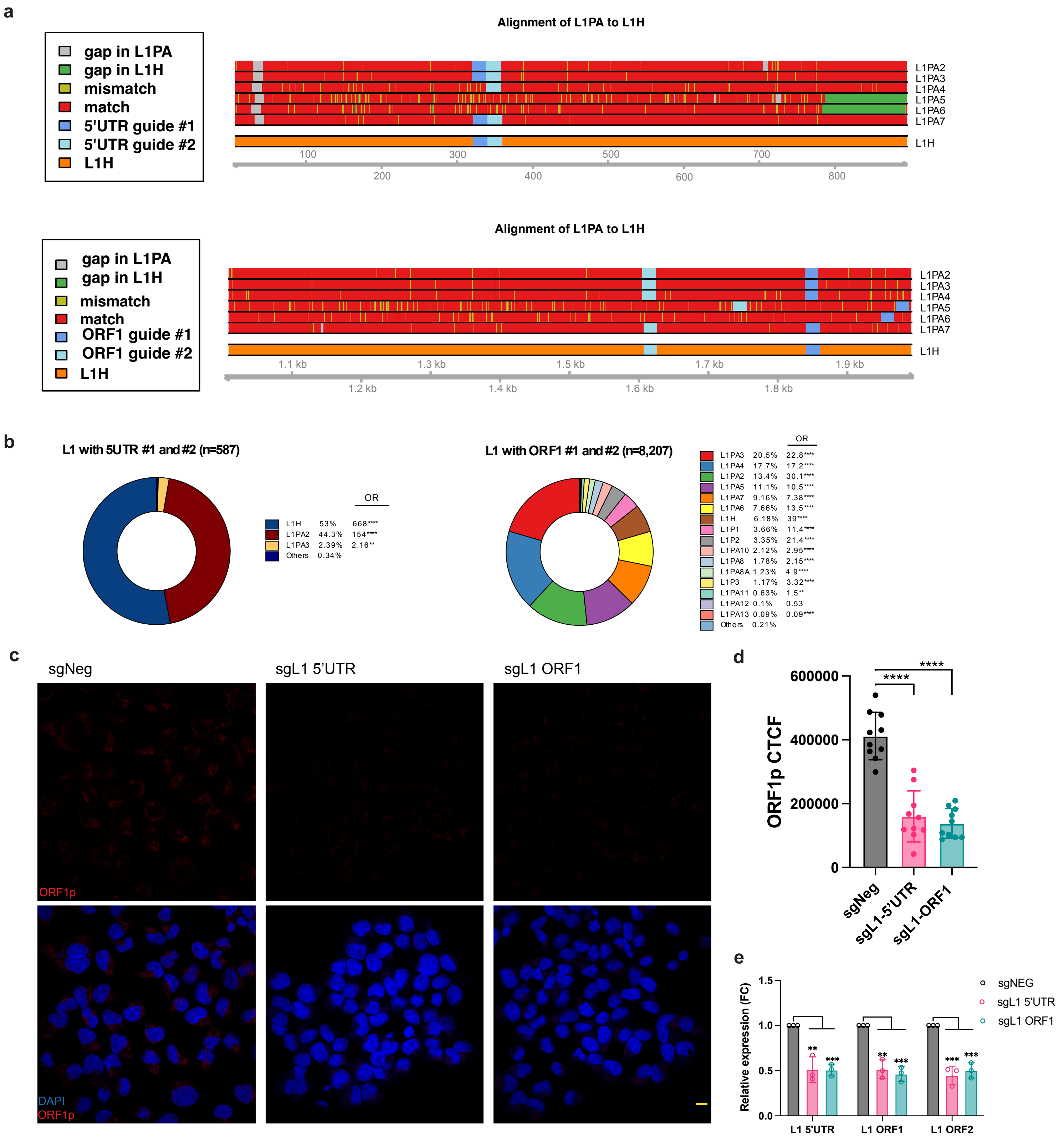
a
Alignment of L1PA to L1H
gap in L1PA
gap in L1H
mismatch
match
5'UTR guide #1
5'UTR guide #2
L1H
L1PA2
L1PA3
L1PA4
L1PA5
L1PA6
L1PA7
L1H
100
300
500
700
200
400
600
800
Alignment of L1PA to L1H
gap in L1PA
gap in L1H
mismatch
match
ORF1 guide #1
ORF1 guide #2
L1H
L1PA2
L1PA3
L1PA4
L1PA5
L1PA6
L1PA7
L1H
1.1 kb
1.3 kb
1.5 kb
1.7 kb
1.9 kb
1.2 kb
1.4 kb
1.6 kb
1.8 kb
b
L1 with 5UTR #1 and #2 (n=587)
OR
L1H 53% 668****
L1PA2 44.3% 154****
L1PA3 2.39% 2.16**
Others 0.34%
L1 with ORF1 #1 and #2 (n=8,207)
OR
L1PA3 20.5% 22.8****
L1PA4 17.7% 17.2****
L1PA2 13.4% 30.1****
L1PA5 11.1% 10.5****
L1PA7 9.16% 7.38****
L1PA6 7.66% 13.5****
L1H 6.18% 39****
L1P1 3.66% 11.4****
L1P2 3.35% 21.4****
L1PA10 2.12% 2.95****
L1PA8 1.78% 2.15****
L1PA8A 1.23% 4.9****
L1P3 1.17% 3.32****
L1PA11 0.63% 1.5**
L1PA12 0.1% 0.53
L1PA13 0.09% 0.09****
Others 0.21%
c
sgNeg
sgL1 5'UTR
sgL1 ORF1
ORF1p
DAPI
ORF1p
d
ORF1p CTCF
600000
400000
200000
0
****
****
sgNeg
sgL1-5'UTR
sgL1-ORF1
e
Relative expression (FC)
1.5
1.0
0.5
0.0
**
***
**
***
***
***
L1 5'UTR
L1 ORF1
L1 ORF2
sgNEG
sgL1 5'UTR
sgL1 ORF1

**Supplementary Fig. 8. CRISPR-mediated knock out/down of L1 elements.**

**a,** The plots display the tracks of the alignment of full-length L1PA2-L1PA7 elements to L1H using the Smith-Waterman pairwise local alignment method. The alignment results are visually represented, marking the locations of guides, gaps inserted in each of the L1H and L1P elements, as well as mismatches and matches. The top plot exhibits the initial 900 base pairs of the sequence alignment, highlighting the 5'UTR guides, while the bottom plot illustrates the subsequent 1KB of the sequence alignment, which shows the ORF1 guides marking.

**b,** The donut plots show the counts of LINE-1 elements in the whole genome that include either 5'UTR guide #1 or #2 (left donut plot) and either ORF1 #1 or #2 (right donut plot). The odds ratio (OR) and p-value were calculated using the Fisher exact test by comparing the counts of LINE-1 subfamilies that include the guides with the counts of these subfamilies in the whole genome. $^{**}p<0.01$ and $^{****}p<0.0001$. The p-values of the 5UTR #1 and #2 donut plot (left) are <2.2e-16 for L1HS and L1PA2, and 8.71e-03 for L1PA3.

**c,** Confocal microscopy of anti-ORF1p immunofluorescence in BT16 cell line with sgRNAs against negative control, L1-5’UTR, or L1-ORF1. ORF1p was stained in red, and nuclei were stained in blue (DAPI). Scale bars, 10 µm.

**d,** Quantification of ORF1p expression performed by measuring corrected total cell fluorescence (CTCF), using ImageJ. Data are mean ± SD of 10 data points; P-value is calculated by unpaired t test with Welch's correction (two-tailed).

**e,** The expression of L1 elements in BT16 cell line with sgRNAs against negative control, L1-5’UTR, or L1-ORF1 was measured by quantitative real-time PCR. Data are mean ± SD of three independent experiments; P-value is calculated by multiple unpaired t tests (two-tailed) followed by correction for multiple testing. $*p<0.05$; $**p<0.01$; $***p<0.001$.

Source data are provided as a Source Data file.

**Supplementary Table 1. sgRNA sequences used for knockout experiment.**

| Target name | sgRNA sequence |
|---|---|
| sgLuc | AATTGTCTTGTCCCTATCGA |
| sgcGAS | GACTCGGTGGGATCCATCG |
| sgMAVS | TACTTCATTGCGGCACTGA |
| sgEZH2 #1 | TGCGACTGAGACAGCTCAAG |
| sgEZH2 #2 | TTATGATGGGAAAGTACACG |
| sgL1-5'UTR #1 | TGGTGCGCCGTTTCTTAAGC |
| sgL1-5'UTR #2 | ACGAGACTATATCCCACACC |
| sgL1-ORF1 #1 | GAGCTCTTTTAGGGCAGGCC |
| sgL1-ORF1 #2 | TTAAGGGCAGCCAGAGAGAA |

**Supplementary Table 2. qPCR primers sequences used in this study.**

| Gene name | | Sequence (5'-3') |
|---|---|---|
| RPLP0 | Forward | CAGACAGACACTGGCAACA |
| | Reverse | ACATCTCCCCCTTCTCCTT |
| ISG15 | Forward | GCCTCAGCTCTGACACC |
| | Reverse | CGAACTCATCTTTGCCAGTACA |
| IRF7 | Forward | GTGGACTGAGGGCTTGTAG |
| | Reverse | TCAACACCTGTGACTTCATGT |
| DDX58 | Forward | CCAGCATTACTAGTCAGAAGGAA |
| | Reverse | CACAGTGCAATCTTGTCATCC |
| OASL | Forward | GCAGAAATTTCCAGGACCAC |
| | Reverse | CCCATCACGGTCACCATTG |
| OAS1 | Forward | AGCACTGGTACCAAAATTGTAAGAAG |
| | Reverse | CCTCGCTCCCAAGCATAGAC |
| INFB1 | Forward | GTCAGAGTGGAAATCCTAAG |
| | Reverse | ACAGCATCTGCTGGTTGAAG |
| INFL1 | Forward | GCCTCCTCACGCGAGACCTC |
| | Reverse | GGAGTAGGGCTCAGCGCATA |
| INFL3 | Forward | TGGCCCTGACGCTGAAGGTT |
| | Reverse | CGTGGGCTGAGGCTGGATAC |
| L1-ORF1 | Forward | TCAAAGGAAAGCCCATCAGACTA |
| | Reverse | TGGCCCCCACTCTCTTCT |
| L1-5UTR | Forward | ACGGAATCTCGCTGATTGCTA |
| | Reverse | AAGCAAGCCTGGGCAATG |
| L1-ORF2 | Forward | AAATGGTGCTGGGAAAACTG |
| | Reverse | GCCATTGCTTTTGGTGTTTT |
| Alu | Forward | GGTGAAACCCCGTCTCTACT |
| | Reverse | GGTTCAAGCGATTCTCCTGC |
| GAPDH | Forward | CTCTCTGCTCCTCCTGTTCG |
| | Reverse | TTTCTCTCCGCCCGTCTTC |
| EV AluJb-AluSz repeat1 | Forward | TCTGCCTTTCCTTGCACTGC |
| | Reverse | GAGGTCGAGGTTACAGTGAGC |
| EV AluJb-AluSz repeat2 | Forward | AACATCAACACACGCCATGC |
| | Reverse | CTCAAATGATCCGTCCGCCT |
| EV AluSp-AluSp repeat1 | Forward | AAAATCGGTTGGGTGCAGTG |
| | Reverse | TGGCCAGACTGGTCTTGAAC |
| EV AluSp-AluSp repeat2 | Forward | GCTTCACGCCTTCGGTCATT |
| | Reverse | TGTGGTGAGCCGAGATTGTG |

| | | |
|---|---|---|
| EV AluSz-AluSx1 repeat1 | Forward | CCTCAAGTGATCCACCCACC |
| | Reverse | CTGTGGCTCTGTGACCTCTT |
| EV AluSz-AluSx1 repeat2 | Forward | TTTGAGAACAGCCTGGCCAA |
| | Reverse | AGGATTACAAGTGCCCTGCC |
| AluJo-AluYm1 repeat1 | Forward | AGCACCTGTATGGAGGTCCT |
| | Reverse | AGACTCCAGCCTGGGTAACA |
| AluJo-AluYm1 repeat2 | Forward | CAGATAGGCCGGGCGAGG |
| | Reverse | TCCATCTCCTGACCTCGTGA |
| AluSx-AluJb repeat1 | Forward | GGTGACACAGCCAGACTCTG |
| | Reverse | CATCTGGGGAAACTGAGGCA |
| AluSx-AluJb repeat2 | Forward | GCCCAGGCTGATCTTGAACT |
| | Reverse | GTAGCTGGGTATGGTGGCTG |
| AluJb-AluSg repeat1 | Forward | GCTCCAACAACCTTGAATTCCA |
| | Reverse | TGTGCCATTGCCCTCTAGC |
| AluJb-AluSg repeat2 | Forward | GCACTTTACTAATCCTCATGGCAC |
| | Reverse | TCTCGAACTCCTGACCTCGT |
| AluJb-AluY repeat1 | Forward | GGCTGGTCTTGAATCCTGGG |
| | Reverse | CCAAGTCAGCCAGGAATGGT |
| AluJb-AluY repeat2 | Forward | TGGGCAACAGAGCAAGACTC |
| | Reverse | TCATGTGGGCCTGAAGCATT |

**Supplementary Table 3. Primary antibodies used in this study.**

| Target | Company | Catalog#/Clone | Dilution |
|---|---|---|---|
| γH2AX-S139 | MilliporeSigma | 05-636/JBW301 | IF: 1/500<br>WB: 1/1000 |
| cGAS | Cell Signaling | 15102/D1D3G | IF: 1/200<br>WB: 1/1000 |
| pSTAT1-Y701 | Cell Signaling | 9167/58D6 | WB: 1/1000 |
| IRF7 | Thermo Fisher | 51-3300 | WB: 1/1000 |
| ssDNA | MilliporeSigma | MAB3299 | IF: 1/100 |
| pSTING-S366 | Cell Signaling | 19781/D7C3S | WB: 1/2000 |
| pTBK1-S172 | Cell Signaling | 5483/D52C2 | WB: 1/1000 |
| dsDNA | Santa Cruz | HYB331-01 | IF: 1/200 |
| MAVS | Abcam | ab89825 | WB: 1/3000 |
| dsRNA | Scicons | 10020500/K1 | IF: 1/200 |
| VDAC | Cell Signaling | 4661/D73D12 | WB: 1/1000 |
| β-Actin | Santa Cruz | sc-47778 | WB: 1/3000 |
| **H3K27me3** | Diagenode | C15410069 | IF: 1/500 |
| **H3K27me3** | Abcam | ab6002 | IF: 1/500<br>WB: 1/1000 |
| **STING** | Abcam | ab181125 | IF: 1/100 |
| **LINE-1 ORF1p** | MilliporeSigma | MABC1152 | IF: 1/100<br>WB: 1/1000 |
| **Vinculin** | Cell Signaling | 13901S | WB: 1/1000 |
| **α-Tubulin** | MilliporeSigma | T9026 | WB: 1/1000 |
