# Supplementary material for: Inhibiting EZH2 targets atypical teratoid rhabdoid tumor by triggering viral mimicry via both RNA and DNA sensing pathways
Source: Nat Commun. 2024 Oct 29;15:9321. doi: 10.1038/s41467-024-53515-8 (PMC11522499; doi:10.1038/s41467-024-53515-8)
Supplement: Supplementary file 3 — Reporting Summary [file 41467_2024_53515_MOESM3_ESM.pdf]

nature portfolio

Corresponding author(s): Dr. Daniel D. De Carvalho

Last updated by author(s): Sep 6, 2024

Reporting Summary

Nature Portfolio wishes to improve the reproducibility of the work that we publish. This form provides structure for consistency and transparency in reporting. For further information on Nature Portfolio policies, see our Editorial Policies and the Editorial Policy Checklist.

Statistics

For all statistical analyses, confirm that the following items are present in the figure legend, table legend, main text, or Methods section.

n/a	Confirmed	
☐	☒	The exact sample size (*n*) for each experimental group/condition, given as a discrete number and unit of measurement
☐	☒	A statement on whether measurements were taken from distinct samples or whether the same sample was measured repeatedly
☐	☒	The statistical test(s) used AND whether they are one- or two-sided<br>*Only common tests should be described solely by name; describe more complex techniques in the Methods section.*
☒	☐	A description of all covariates tested
☐	☒	A description of any assumptions or corrections, such as tests of normality and adjustment for multiple comparisons
☐	☒	A full description of the statistical parameters including central tendency (e.g. means) or other basic estimates (e.g. regression coefficient) AND variation (e.g. standard deviation) or associated estimates of uncertainty (e.g. confidence intervals)
☐	☒	For null hypothesis testing, the test statistic (e.g. *F*, *t*, *r*) with confidence intervals, effect sizes, degrees of freedom and *P* value noted<br>*Give P values as exact values whenever suitable.*
☒	☐	For Bayesian analysis, information on the choice of priors and Markov chain Monte Carlo settings
☒	☐	For hierarchical and complex designs, identification of the appropriate level for tests and full reporting of outcomes
☐	☒	Estimates of effect sizes (e.g. Cohen's *d*, Pearson's *r*), indicating how they were calculated

Our web collection on statistics for biologists contains articles on many of the points above.

Software and code

Policy information about availability of computer code

Data collection	Confocal microscopy was performed using Leica SP8 Confocal Microscope. Western blotting data was collected by ImageLab v6.0 (Bio-Rad).
Data analysis	Statistical analyses for cell biological experiments were performed with GraphPad Prism (version 8, and 9).<br>RNA-seq analysis: trim_galore (v.0.5.0), STAR aligner program (v. 2.5.2), picard program (v.1.9.1), samtools (v.1.9), featureCounts (v. 1.6.2), python (v. 2.7.15) and R package (v.4.1.0).<br>CUT&RUN analysis: fastp version 0.19.5, bowtie2 (v. 2.3.5), genomecov command in the bedtools suite (v. 2.27.1), deeptools (v. 3.2.1), python (v. 2.7.15).<br>Gene Set Enrichment Analysis (GSEA) was conducted using the GSEA software available at http://www.broad.mit.edu/GSEA.

For manuscripts utilizing custom algorithms or software that are central to the research but not yet described in published literature, software must be made available to editors and reviewers. We strongly encourage code deposition in a community repository (e.g. GitHub). See the Nature Portfolio guidelines for submitting code & software for further information.

Data

Policy information about availability of data

All manuscripts must include a data availability statement. This statement should provide the following information, where applicable:

- Accession codes, unique identifiers, or web links for publicly available datasets
- A description of any restrictions on data availability
- For clinical datasets or third party data, please ensure that the statement adheres to our policy

Raw sequencing data of RNA-seq and CUT&RUN have been deposited at the Gene Expression Omnibus (GEO) (https://www.ncbi.nlm.nih.gov/geo) under the accession number GSE213250.

Research involving human participants, their data, or biological material

Policy information about studies with human participants or human data. See also policy information about sex, gender (identity/presentation), and sexual orientation and race, ethnicity and racism.

Reporting on sex and gender	*Use the terms sex (biological attribute) and gender (shaped by social and cultural circumstances) carefully in order to avoid confusing both terms. Indicate if findings apply to only one sex or gender; describe whether sex and gender were considered in study design; whether sex and/or gender was determined based on self-reporting or assigned and methods used. Provide in the source data disaggregated sex and gender data, where this information has been collected, and if consent has been obtained for sharing of individual-level data; provide overall numbers in this Reporting Summary. Please state if this information has not been collected. Report sex- and gender-based analyses where performed, justify reasons for lack of sex- and gender-based analysis.*
Reporting on race, ethnicity, or other socially relevant groupings	*Please specify the socially constructed or socially relevant categorization variable(s) used in your manuscript and explain why they were used. Please note that such variables should not be used as proxies for other socially constructed/relevant variables (for example, race or ethnicity should not be used as a proxy for socioeconomic status). Provide clear definitions of the relevant terms used, how they were provided (by the participants/respondents, the researchers, or third parties), and the method(s) used to classify people into the different categories (e.g. self-report, census or administrative data, social media data, etc.) Please provide details about how you controlled for confounding variables in your analyses.*
Population characteristics	The cohort consists of 25 patients where 52% are Female (n=13), 44% are Male (n=11) and one patient with no reported status.
Recruitment	The cohort is a retrospective cohort collected through the Rare Brain Tumor Consortium and Registry (www.rarebraintumorconsortium.ca) from its global partners with informed consent as per protocols approved by the Research Ethics Board of the Hospital for Sick Children..
Ethics oversight	the Hospital for Sick Children

Note that full information on the approval of the study protocol must also be provided in the manuscript.

Field-specific reporting

Please select the one below that is the best fit for your research. If you are not sure, read the appropriate sections before making your selection.

☒ Life sciences ☐ Behavioural & social sciences ☐ Ecological, evolutionary & environmental sciences

For a reference copy of the document with all sections, see nature.com/documents/nr-reporting-summary-flat.pdf

Life sciences study design

All studies must disclose on these points even when the disclosure is negative.

Sample size	Sample sizes for the in vitro experiments were determined based on prior knowledge on variation with similar assays, and/or from sizes generally used in the field. Specific sample sizes and the number of independent experiments for each study is available within the figures, figure legends, or method section.
Data exclusions	No data were excluded from analysis or reporting
Replication	All experiments were carried out independently following the descriptions provided in the figure legends or were biologically replicated a minimum of 2-3 times. The p-values were calculated based on measurements obtained from experiments conducted independently at least three times. Representative images or results with similar numerical values were selected, and these were replicated in at least two independent biological experiments.
Randomization	The quantification of immunofluorescence (IF) data involved random regions on the slide. No other randomization was conducted as the remaining experiments were carried out in vitro using eukaryotic cell lines. Data were compared or collected under identical conditions where

randomness was unnecessary.

Blinding

Samples were blinded during the analysis of practically feasible experiments (Fig. 2b; Fig. 4b, c, d; Fig. 6b). However, the majority of experiments were not conducted under blinded conditions, as the collected data were quantitative and not subjective. Most data were derived from at least 2 to 3 biologically independent experiments, replicated by different investigators, or validated to yield consistent results through various experimental methods.

Reporting for specific materials, systems and methods

We require information from authors about some types of materials, experimental systems and methods used in many studies. Here, indicate whether each material, system or method listed is relevant to your study. If you are not sure if a list item applies to your research, read the appropriate section before selecting a response.

Materials & experimental systems

n/a	Involved in the study
☐	☒ Antibodies
☐	☒ Eukaryotic cell lines
☒	☐ Palaeontology and archaeology
☒	☐ Animals and other organisms
☒	☐ Clinical data
☒	☐ Dual use research of concern
☒	☐ Plants

Methods

n/a	Involved in the study
☒	☐ ChIP-seq
☐	☒ Flow cytometry
☒	☐ MRI-based neuroimaging

Antibodies

Antibodies used

γH2AX-S139 MilliporeSigma 05-636/JBW301 IF: 1/500
WB: 1/1000
cGAS Cell Signaling 15102/D1D3G IF: 1/200
WB: 1/1000
pSTAT1-Y701 Cell Signaling 9167/58D6 WB: 1/1000
IRF7 Thermo Fisher 51-3300 WB: 1/1000
ssDNA MilliporeSigma MAB3299 IF: 1/100
pSTING-S366 Cell Signaling 19781/D7C3S WB: 1/2000
pTBK1-S172 Cell Signaling 5483/D52C2 WB: 1/1000
dsDNA Santa Cruz HYB331-01 IF: 1/200
MAVS Abcam ab89825 WB: 1/3000
dsRNA Scicons 10020500/K1 IF: 1/200
VDAC Cell Signaling 4661/D73D12 WB: 1/1000
β-Actin Santa Cruz sc-47778 WB: 1/3000
H3K27me3 Diagenode C15410069 IF: 1/500
H3K27me3 Abcam ab6002 IF: 1/500
WB: 1/1000
STING Abcam ab181125 IF: 1/100
LINE-1 ORF1p MilliporeSigma MABC1152 IF: 1/100
WB: 1/1000
Vinculin Cell Signaling 13901S WB: 1/1000
α-Tubulin MilliporeSigma T9026 WB: 1/1000

Validation

The experimental design incorporated relevant positive and negative controls to confirm the specificity of the antibodies.

Eukaryotic cell lines

Policy information about cell lines and Sex and Gender in Research

Cell line source(s)

CHLA02-ATRT (ATCC, Cat# CRL-3020) and CHLA05-ATRT (ATCC, Cat# CRL-3037) were obtained from American Type Culture Collection (ATCC, Teddington, UK). BT12 and BT16 were kind gifts from Dr. Annie Huang at Hospital for Sick Children.

Authentication

A 10-locus STR authentication panel (GenePrint 10 System, Promega) was employed.

Mycoplasma contamination

Cells were routinely tested for and found to be mycoplasma free.

Commonly misidentified lines
(See ICLAC register)

No misidentified cell lines were used.

Plants

Seed stocks

Report on the source of all seed stocks or other plant material used. If applicable, state the seed stock centre and catalogue number. If plant specimens were collected from the field, describe the collection location, date and sampling procedures.

Novel plant genotypes

Describe the methods by which all novel plant genotypes were produced. This includes those generated by transgenic approaches, gene editing, chemical/radiation-based mutagenesis and hybridization. For transgenic lines, describe the transformation method, the number of independent lines analyzed and the generation upon which experiments were performed. For gene-edited lines, describe the editor used, the endogenous sequence targeted for editing, the targeting guide RNA sequence (if applicable) and how the editor was applied.

Authentication

Describe any authentication procedures for each seed stock used or novel genotype generated. Describe any experiments used to assess the effect of a mutation and, where applicable, how potential secondary effects (e.g. second site T-DNA insertions, mosiacism, off-target gene editing) were examined.

Flow Cytometry

Plots

Confirm that:

☒ The axis labels state the marker and fluorochrome used (e.g. CD4-FITC).

☒ The axis scales are clearly visible. Include numbers along axes only for bottom left plot of group (a 'group' is an analysis of identical markers).

☒ All plots are contour plots with outliers or pseudocolor plots.

☒ A numerical value for number of cells or percentage (with statistics) is provided.

Methodology

Sample preparation

Stained cancer cell lines were collected, and single cell suspension was prepared following the protocol from the manufacturer.

Instrument

Stained cells were analyzed using a BD LSR II Flow Cytometer.

Software

Data were analyzed using FlowJo.

Cell population abundance

Over 95% on sorted cells, determined by flow cytometry on cells post sorting.

Gating strategy

Doublets were removed by FSC-A/FSC-W. For CTV assay, live cells were gated through FSC-A/SSC-A.

☐ Tick this box to confirm that a figure exemplifying the gating strategy is provided in the Supplementary Information.
